# Preparation and Optimization of Optical pH Sensor Based on Sol-Gel

**DOI:** 10.3390/s18103195

**Published:** 2018-09-21

**Authors:** Jianxin Zhang, Lei Zhou

**Affiliations:** Faculty of Mechanical Engineering & Automation, Zhejiang Sci-Tech University, Hangzhou 310018, China; leizjlg@foxmail.com

**Keywords:** sol-gel technique, optical pH sensor, indicator, sensor-sensitive membrane

## Abstract

Making use of the sol-gel technique, an optical pH sensor was prepared, which was made from an organic carrier with four indictors including congo red, bromophenol blue, cresol red, and chlorophenol red, cross-linked by tetraethyl orthosilicate (TEOS) and cellulose acetate. The actual detection range of the optical pH sensor is 2.5–11.0. The optimal ratio of ethyl orthosilicate, absolute ethanol, deionized water, and hydrochloric acid in glue precursor of the sensor-sensitive membrane was explored. The orthogonal experiment was designed to optimize the dosage of cellulose acetate, *N*,*N*-dimethylformamide (DMF), indicator, hydrochloric acid, and precursor glue in preparing the sensor-sensitive membrane. The linearity, measurement accuracy, repeatability, stability, and response time of the prepared pH sensor were tested. The measurement results were analyzed using a support vector machine and linear regression. The experimental results show that the optical pH sensor has a measurement accuracy of up to 0.2 pH and better stability and repeatability than the traditional pH glass electrode.

## 1. Introduction

The pH value is a measurement of the hydrogen ion concentration in a solution. In environmental [1], industrial, pharmaceutical [2], and food industries [3], the pH value is a very important parameter. Currently, the pH glass electrode is widely used for the measurement of pH values [4], as it has the advantages of a wide measuring range and high accuracy [5]. However, the pH glass electrode has a short lifetime [6] and is susceptible to interference from electromagnetic wave and radio frequency; it is also easily contaminated or poisoned while being used in harsh environments (high temperature, high pressure, high corrosion, etc.), which leads to unstable or invalid measuring results [7]. Therefore, various research on non-glass pH sensors [8,9] is gradually becoming a hot spot. Optical pH sensors recently drew increased attention from researchers and users for its stable measurement signal, ease of transport, strong anti-electromagnetic-interference properties, pollution-free nature, and other advantages [10,11].

An optical pH sensor is mainly composed of a pH indicator and an organic carrier. Its principle of measurement is to utilize the acid-based indicator to mark the hydrogen ions in the solution [12]. The interaction between the indicator and the hydrogen ions changes the structure of the indicator molecule, varying the optical properties of the optical pH sensor (absorption rate, reflectance, fluorescence, refractive index, color, etc.). By measuring the variations in optical characteristics, the pH value of the solution can be determined. At present, the more commonly used indicator types are fluorescent acrylamide [13], thymol blue [14], cresol red [15], bromophenol blue [16], and chlorophenol red [17], and a new nanometer-based indicator is also under development. In general, the indicator should be immobilized on an organic carrier, such as a polymer, agarose, hydrogel, or cellulose, via methods including adsorption, chemical bonding, and the sol-gel method [18]. The carrier can then be fixed on the probe to form an optical pH sensor for sensing pH variation for long hours in a solution.

The optical detection method of the optical pH sensor has a significant impact on its accuracy. Current optical detection methods generally sense the solution’s pH value by detecting the change in optical parameters of the solution [19]. Such a method is easily affected by environmental factors, limiting its measurement range and accuracy. Some other methods measure the effect of pH on the incident light spectrum, which requires the use of a spectrometer to obtain spectral characteristics and to develop the spectral model to analyze the pH value. This kind of method has good environmental adaptability, and high measurement accuracy.

In this paper, an optical pH sensor based on sol-gel was prepared by embedding four indicators (congo red, bromophenol blue, cresol red, and chlorophenol red) into an organic carrier formed by the cross-linking of tetraethyl orthosilicate (TEOS) and cellulose acetate. The optimized parameters for the usage of materials, including cellulose acetate, *N*,*N*-dimethylformamide (DMF), indicators, hydrochloric acid, and precursor glue, were determined by experiments.

## 2. Experiments

### 2.1. Chemical and Instruments

TEOS and cellulose acetate (C_6_H_7_O_2_(OH)_3−m_(OOCCH_3_)_m_; m = 0–3) were purchased from Tianjin Kemi Europe Chemical Reagent Co., Ltd. Absolute ethanol (C_2_H_5_OH) was purchased from Hangzhou Gao Jing Fine Chemical Co., Ltd (Hangzhou, China). Congo red indicator (C_32_H_22_N_6_Na_6_O_6_S_2_, crystallization) and DMF were purchased from Aladdin Reagent Company, China. Cresol red (C_21_H_18_O_5_S, crystallization), bromophenol blue (C_19_H_10_Br_4_O_5_S, crystallization), and chlorophenol red (C_19_H_12_Cl_2_O_5_S, crystallization) were purchased from Shanghai Macklin Biochemical Technology Co., Ltd. The deionized water and dilute hydrochloric acid (HCl 2 M) used in the experiment were laboratory-made. Sodium tetraborate, potassium hydrogen phthalate, and mixed phosphate were purchased from Shanghai Shengke Equipment Co., Ltd. Citric acid (C_6_H_8_O_7_·H_2_O), sodium citrate (C_6_H_5_Na_3_O_7_·2H_2_O), sodium carbonate (Na_2_CO_3_), and sodium bicarbonate (NaHCO_3_) were purchased from Aladdin Reagent Company (Shanghai, China).

The instruments used in our experiments were as follows: (1) water bath heating device(Shanghai Leton Industrial Co., Ltd. Shanghai, China), used to control the temperature (0–101 °C) during the preparation of the precursor glue; (2) electronic balance(Henan Hengxin Instrument Equipment Co., Ltd. Zhengzhou, China), used to gauge the quantity of the indicator and other materials; (3) mixer(Shanghai Meiyingpu Instrument & Meter Manufacturing Co., Ltd. Shanghai, China), used to prepare the precursor glue; (4) CNC ultrasonic cleaner(Kunshan Ultrasonic Instrument Co., Ltd. Kunshan, China), used to dissolve the cellulose acetate; (5) vacuum oven, used to dry the sensitive film glue; and (6) pH meter, used to configure the pH of the buffer configuration.

### 2.2. Preparation and Optimization of Sol-Gel pH-Sensitive Membrane

#### 2.2.1. Principle of Modified Sol-Gel Preparation

The sol-gel organic carrier prepared in this work was composed of a network structure formed from TEOS hydrolyzed, condensed, and cross-linked with cellulose acetate. The chemical reactions involved in the preparation are described below.

The TEOS hydrolysis reaction equation [20] was as follows:

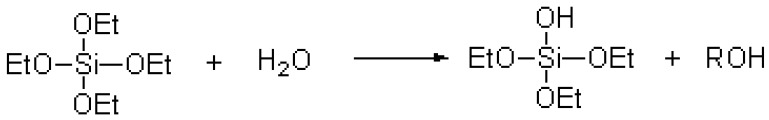
;
(1)

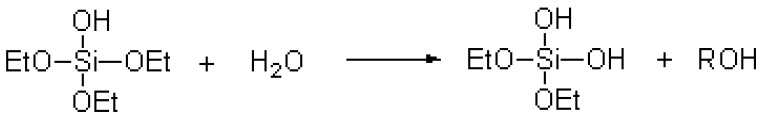
;
(2)

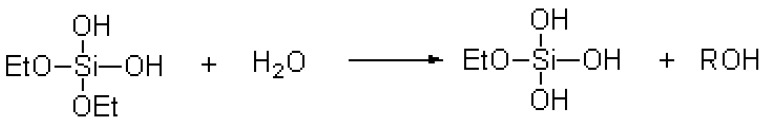
;
(3)

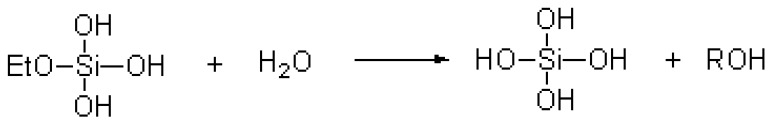
.
(4)

The TEOS polycondensation reaction equation [20] was as follows:

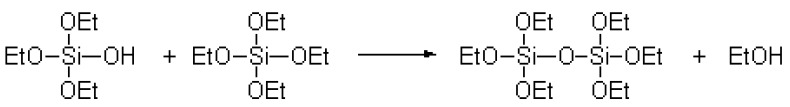
;
(5)

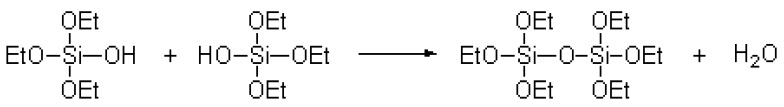
.
(6)

The total reaction equation [20] was as follows:

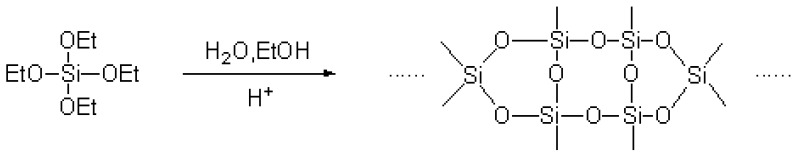
.
(7)

The TEOS and cellulose acetate cross-linking reaction [20] was as follows:

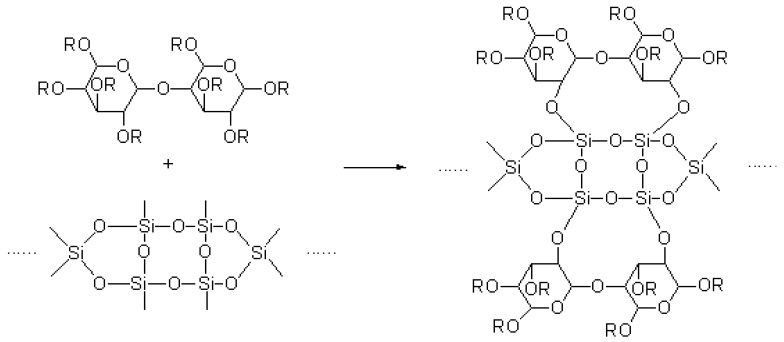
.
(8)

#### 2.2.2. Preparation of Sol-Gel pH-Sensitive Membranes

The preparation steps of the pH-sensitive membranes were as follows:

(1) A certain amount of cellulose acetate was dissolved in DMF, stirred thoroughly, and placed in an ultrasonic cleaner for 2 min, before the solution was placed on a magnetic stirrer and continued stirring at 60 °C for 5 h.

(2) A mixed solution was prepared using a certain volume of TEOS, absolute ethanol, hydrochloric acid, and deionized water with a fixed molar ratio.

(3) The solutions prepared in (1) and (2) were mixed, and the four indicators (congo red, bromophenol blue, cresol red, and chlorophenol red) were added; the mixed solution was placed in an ultrasonic cleaner to vibrate for 5 min so as to dissolve the indicator completely.

(4) The mixed solution obtained in (3) was stirred at 50 °C on a magnetic stirrer until a homogeneous transparent sol was formed, and the sol was left for 12 h to allow foaming and aging.

(5) The sol was dried in the vacuum oven at 70 °C.

#### 2.2.3. Optimization of Sol-Gel pH-Sensitive Membrane

While preparing the sol–gel pH-sensitive membranes, the reaction conditions for the sol-gel precursor and the amounts of each component of the sensitive membrane, including cellulose acetate, DMF, indicators, hydrochloric acid, and precursor glue needed to be optimally determined.

(i) Reaction Conditions of Sol Precursor

The hydrolysis and polycondensation reaction of TEOS under acid catalysis was mainly influenced by several factors, such as the amounts of ethanol, water, and hydrochloric acid, as well as the dropping speed and the temperature.

(1) Amount of Ethanol

**Figure 1:**
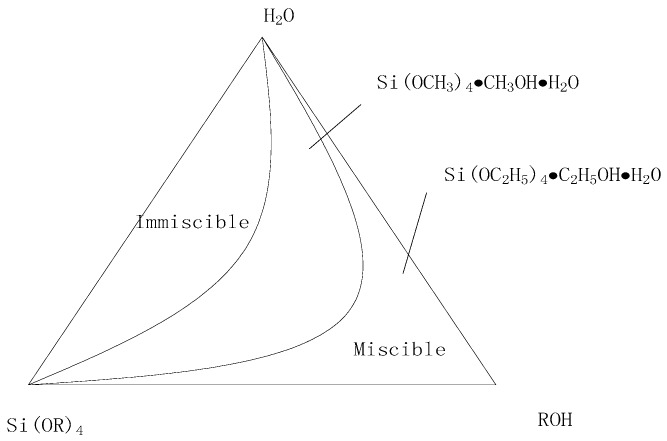
Ternary miscibility diagram.

The sol-gel precursor solution was prepared using TEOS as the raw material. Because of the limited solubility of TEOS in water, a certain amount of co-solvent was needed. The co-solvent needed to be soluble with TEOS and water; as such, ethanol was chosen as the co-solvent. The ternary miscibility diagram of TEOS, C_2_H_5_OH, and H_2_O is shown in Figure 1. From Figure 1, it can be concluded that the addition of less ethanol would lead the ternary solution system to be immiscible, but the addition of more ethanol would immobilize the hydrolysis reaction of the system. In this paper, the molar ratio of ethanol to TEOS was 2:1.

(2) Amount of Water

**Figure 2:**
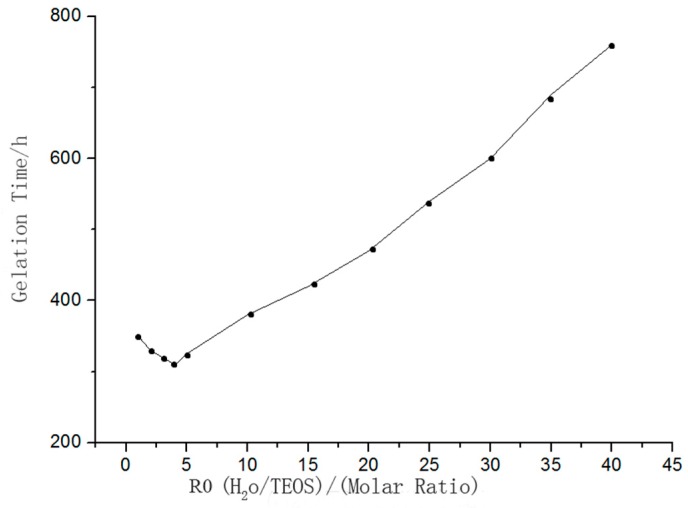
Relationship between water–silica ratio and system gelation time.

When the molar ratio (R0) of water to TEOS was less than 4, the gelation time decreased with increasing R0. TEOS could not be fully hydrolyzed while R0 was less than 4. As R0 increased, so did the concentration of Si–OH groups formed after hydrolysis of TEOS. At this time, the polycondensation reaction proceeded more quickly which decreased the gelation time. On the contrary, if R0 was more than 4, TEOS was be completely hydrolyzed and the concentration of Si–OH groups formed after hydrolysis of TEOS decreased with increasing R0. Hence, the reaction between Si–OH groups through collision was attenuated, resulting in an increased gelation time. Furthermore, as one of the polycondensation products, more water being added resulted in the polycondensation reaction being hindered. The relationship between the water–silica ratio and the system’s gelation time is shown in Figure 2.

Moreover, the amount of water added also affected the drying process of the sensitive membrane, which aggravated its cracking phenomenon. In our work, the final molar ratio of water to TEOS was 4:1.

(3) Dropping Rate and Amount of Hydrochloric Acid

**Figure 3:**
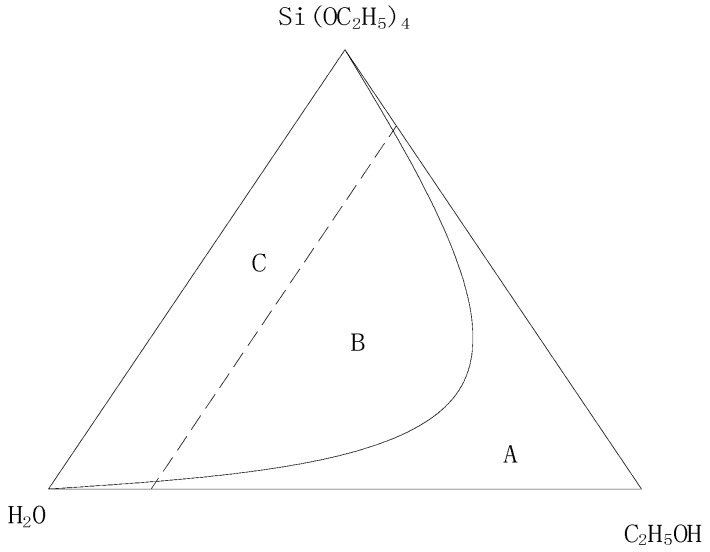
Hydrochloric acid promotes the ternary immiscible area expanding from A to B.

In the sol-gel reaction system, hydrochloric acid can accelerate the rates of hydrolysis and polycondensation, and promote the expansion of TEOS, ethanol, and water from group A in the ternary mixed zone to group B, as shown in Figure 3. In this work, the exact amount of hydrochloric acid was determined using the orthogonal test [21].

During the reaction of hydrochloric acid with TEOS, the titration rate of hydrochloric acid also had a certain influence on the sol-gel.

If the dropping rate of hydrochloric acid is too fast, a white precipitate, instead of the homogeneous gel, is formed in the TEOS solution due to the fast local hydrolysis. By means of dropwise addition, the speed of the hydrolytic polycondensation of TEOS was slowed down so that the reaction could be carried out steadily, which was beneficial for the generation of a homogeneous gel. In this work, deionized water and hydrochloric acid were mixed thoroughly, and the homogeneous precursor solution was prepared by means of dropwise addition.

(4) Temperature

**Figure 4:**
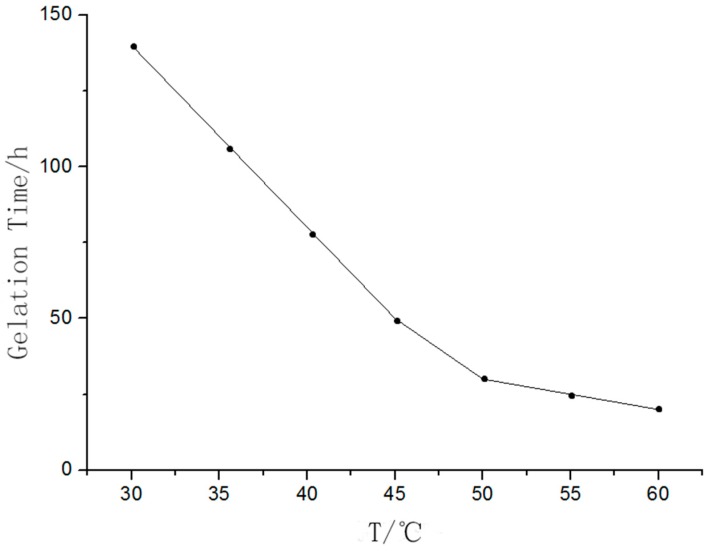
The curve of temperature vs. gelation time.

The relationship between the temperature and gelation time is shown in Figure 4, where it is shown that the gelation time decreased with an increase in temperature.

As the temperature increased, the hydrolytic activity of TEOS was enhanced and the average kinetic energy of the water molecules increased, which increased the probability of collisions between TEOS molecules and water molecules. As a result, the hydrolysis reaction was accelerated, and the gelation time of the mixed solution was reduced. In this work, the temperature was set at 50 °C.

(ii) Optimization of Material Dosage

In this work, the orthogonal test was used to optimize the dosage of the sensor-sensitive membrane materials, including cellulose acetate, DMF, indicator, hydrochloric acid, and precursor glue. With fewer trials, better production conditions or better preparation technology can be obtained using the orthogonal test table (i.e., the optimal ratio of sensitive film materials can be quickly and efficiently obtained).

The orthogonal experiment scheme for the composition ratio of the materials is shown in Table 1, and the optical pH sensors based on sol–gel were prepared accordingly. In total, five factors (cellulose acetate, mixed indicator, DMF, hydrochloric acid, and precursor) were considered and four levels of experiment were conducted. The optimal recipe was determined by comparing the slope of the fitted straight lines of different pH values.

**Figure 5:**
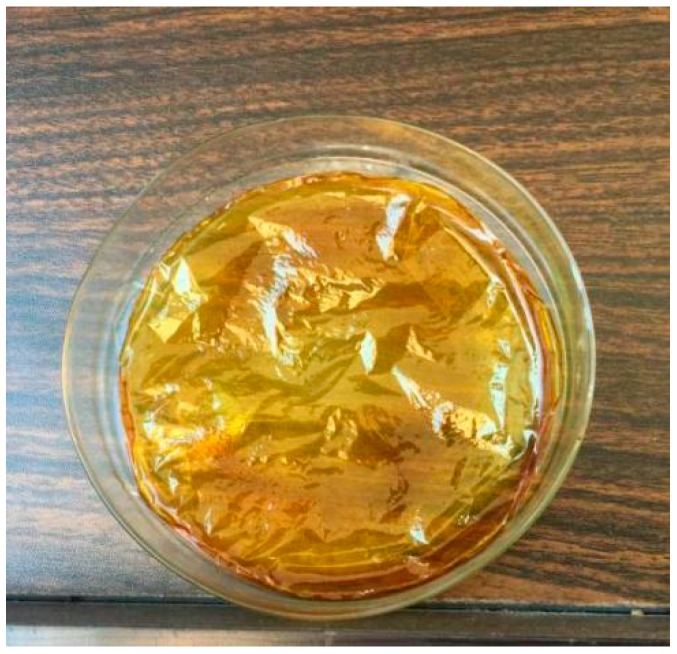
Optimized sensitive membrane (scale bar = 1:2).

The orthogonal experimental results are shown in Table 2, where the slope averages of fitting lines for each level K_i_ (i = 1, 2, 3, 4) are listed at the last four lines. The level corresponding to the max average slope was selected as the optimal value for that factor; thus, $A_{1}B_{3}C_{2}D_{3}E_{2}$ was determined as the optimal combination. However, too much DMF would hinder the drying of the sensitive membrane, leading to easy cracking. The experimental results showed that the preparation of the sensitive membrane with a volume of DMF between 15 mL and 20 mL had a higher successful rate. Hence, the final recipe included 0.1 g of cellulose acetate, 8 g of mixed indicator (congo red, bromophenol blue, cresol red, and chlorophenol red, 2 g each), 15 mL of DMF, 50 µL of hydrochloric acid, and 7.5 mL of precursor. The final optimized sensitive membranes are shown in Figure 5.

## 3. Results and Discussion

### 3.1. Performance Test of the Optical pH Sensor Based on Sol-Gel

The light source (390–680 nm) and spectrometer (USB2000+ from Ocean Optics) were used to measure the spectrum of the sensitive membrane between 300 nm and 800 nm. The optical power meter (BIM-7001) was used to test the performance of the sensor at 532 nm for comparison.

**Figure 6:**
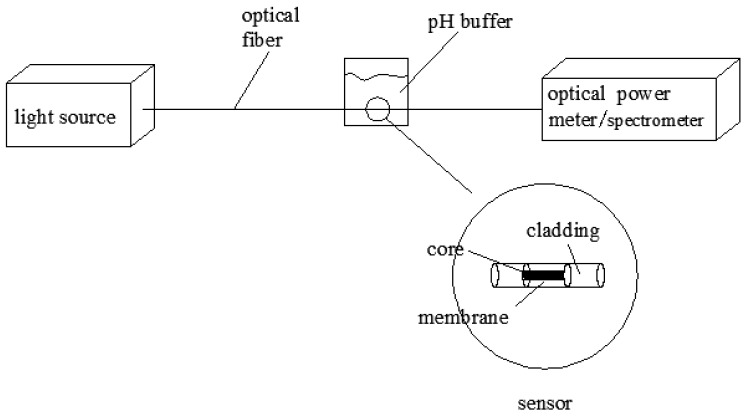
Detection platform.

The prepared sensitive membrane was tested using the test platform shown in Figure 6. The light from the light source passed through the optical pH sensor, where the sensitive membrane was coated on the optical fiber, before being received by an optical power meter or spectrum instrument. The pH buffer solution, with a 0.5 interval, had pH values varying from 2.5 to 11.0, and the power values of the optical power meter were recorded. From the relationship between the power values and the pH values, the linearity, accuracy, stability, repeatability, and response time of the sensor were analyzed and discussed.

#### 3.1.1. Linearity

**Figure 7:**
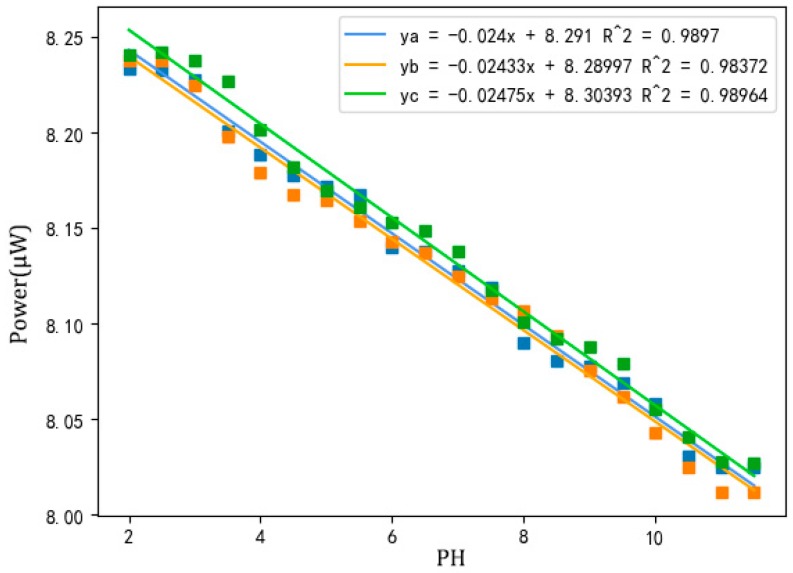
Power–pH curve of three optimized pH sensors.

Three pH sensors prepared under optimized conditions were placed in buffer solutions with different pH values, and measured continuously for 30 s after the value on the power meter was stable. The recording was performed every 10 s. The average value of three measurements was taken as the final value, and the pH value was calculated by fitting a curve as shown in Figure 7.

As can be seen in Figure 7, when the sensor was in a solution with pH ≤ 2.5 or pH ≥ 11.0, there was almost no change in power, which depends on the type of indicator in the sensitive membrane. When the pH value of the solution was between 2.5 and 11.0, the linearity was good and the correlation coefficient was greater than 0.988. As the pH increased, the power value weakened, because the decrease in concentration of hydrogen ions in the solution darkened the sensitive membrane, which increased the absorption of light by the indicator, leading to a decrease in output power [22]. In other published studies, the relationship between pH and power was observed as a sigmoidal curve [23]. However, a good correlation coefficient (0.988) was obtained when using linear fitting. This means that a linear curve is also a suitable approach for the sensor in this paper. In addition, the different intercepts of these three sensors may be related to the cross-linking state of TEOS and cellulose acetate. Different cross-linking conditions affect the embedding of the four indicators in the sensitive membranes, thereby causing the intercept drift; thus, the sensor needs to be calibrated before each use.

#### 3.1.2. Measurement Accuracy

Three standard pH buffer solutions (pH = 4.00, pH = 6.86, pH = 9.18) were measured using the optimized pH sensor. Each sample was measured three times, and the powers were recorded and averaged. The theoretical pH value was calculated according to the curve in Figure 7, and the results obtained are shown in Table 3:

The absolute errors between theoretical pH values calculated in Table 3 and pH values of the three standard buffers were 0.08, 0.24, and 0.13. At pH 6.86, the absolute error was large, which may be caused by the type and dose of the indicators. The sensor worked better in acidic or alkaline solutions than in the near-neutral solution. It is presumed that the measurement accuracy of the sensor can reach up to 0.2 pH.

#### 3.1.3. Stability and Repeatability

**Figure 8:**
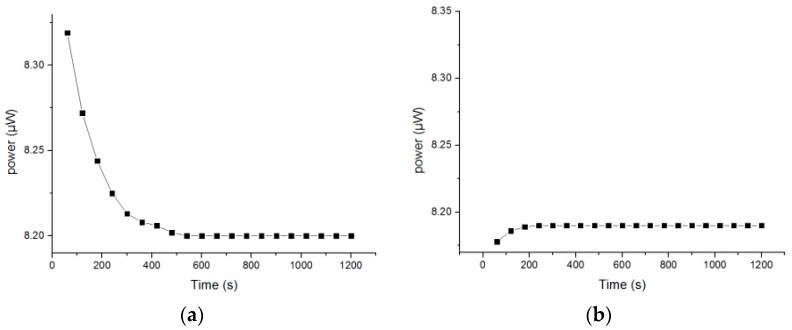
(**a**) First measurement; (**b**) second measurement.

The pH sensor was placed in a buffer solution with a pH of 4.0, and the corresponding power values within 20 min were recorded every minute, as shown in Figure 8a. After testing, the sensor was washed with deionized water three times, before being placed in the same buffer solution to measure for 20 min once more. The change in corresponding power value every minute is shown in Figure 8b.

In Figure 8a, it can be seen that the measured value first rapidly decreased and became stable around 600 s. However, in Figure 8b, at the initial stage, the measured value increased, and gradually became stable at about 200 s, where the standard deviation of the power variation within 20 min was 0.00277, and the relative standard deviation was 0.03911%. Therefore, the sol–gel-based optical pH sensor has good stability. The initial change in Figure 8b was probably due to the washing of the sensor with deionized water.

To study the repeatability of the sensor, the sensor was used to measure the solutions with pH values of 4.0, 6.0, 8.0, and 10.0, each measured three times. The standard deviations and relative standard deviations were calculated in the different pH solutions, and are shown in Table 4.

Table 4 shows that, in the buffer solution with different pH values, the relative standard deviation changed within 1% after measurements were repeated three times. Moreover, after the power meter indicated a stable number, the response value did not vary any more, showing that the sensor has good stability and repeatability. In contrast, when measuring the pH values of different pH buffers with the traditional pH glass electrode, the measurement value struggles for stability, and the indications of the two measurements are potentially inconsistent. Thus, the sensor described here has better stability and repeatability than the traditional glass electrode. However, the stabilization time for the pH sensor in different pH solutions was different, indicating that the sol-gel-based optical pH sensor needs to be active to some extent before measurement.

#### 3.1.4. Response Time

**Figure 9:**
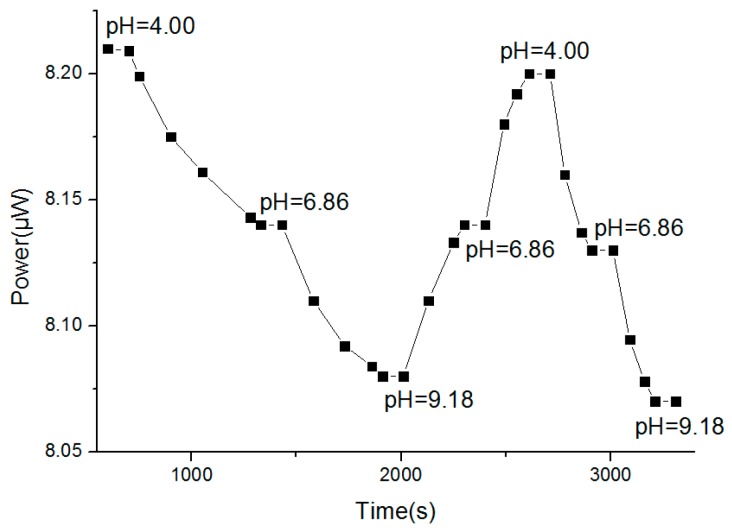
Response time.

The sol-gel-based optical pH sensor was placed in a solution with a pH value of 4.00 at room temperature (25 °C). Once the power meter showed a stable number, the readings of optical power and corresponding response time were recorded. After being washed with deionized water three times, the pH sensor was placed in a solution with a pH of 6.86. This operation was repeated in solutions with pH values of 9.18, 6.86, and 4.00, and the response times are shown as the horizontal ordinate in Figure 9.

From Figure 9, when the pH value changed from 4.00 to 6.86, the response time was about 580 s. When the pH value changed from 6.86 to 9.18, the response time was about 650 s. When the pH value changed from 9.18 to 6.86, the response time was about 430 s. Therefore, the response time showed a declining trend and eventually stabilized near 200 s, implying that the sol–gel-based optical pH sensor requires activation before measurement. The response time after activation was about 200 s which is a median value among existing chemical sensors. The response time mainly depends on the structure of the sensitive membrane material including the pore size, pore size distribution, porosity (which is a multi-pole material formed by the hydrolysis), condensation, and cross-linking with the cellulose acetate of the TEOS. Since the sol-gel pH-sensitive membrane used in this experiment was a porous and three-dimensional network structure, it had good permeability to hydrogen ions, resulting in the proper response time.

### 3.2. Measurement Results Based on Spectrometer

**Figure 10:**
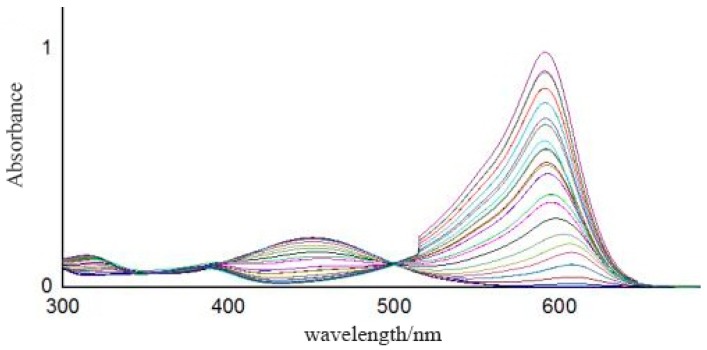
Absorbance spectra.

The full absorbance data between 390 nm and 680 nm of the pH buffer solutions, with pH values ranging from 2.5 to 11.0 with 0.5 intervals, were collected using the spectrometer. As shown in Figure 10, 581 absorbance points in total were selected as valid data. Using a support vector machine (SVM), the absorbance vs. pH model was established. The pH values of three standard pH buffer solutions (pH = 4.00, pH = 6.86, pH = 9.18) were predicted using the established model. As shown in Table 5, compared with the fitting results using the optical meter and absorbance data at a single wavelength (532 nm), the absolute error predicted by the SVM model was much smaller. In terms of accuracy, the results based on full spectral measurement used in this paper are better than those based on single-wavelength optical power. The average absolute error of our prepared pH sensor was 0.164 and better than that of Reference [24], where a full-range optical pH sensor array based on neural networks was designed and the average absolute error was 0.177.

In Figure 10, a higher curve peak around 600 nm suggests a higher solution pH value. It can be seen that the two curves with pH values of 6.5 and 7.0 were close to each other, which corroborates the results in Figure 7 that the absolute error predicted by the SVM model of the near-neutral solution (pH = 6.86) is larger than that of the acidic (pH = 4.00) and alkaline (pH = 9.18) solutions. The acid dissociation constant (p*K*_a_) values of the four indicators in the sensitive membrane are listed in Table 6, where the color of the indicator obviously changes between p*K*_a_ − 1 and p*K*_a_ + 1 [23]. Therefore, according to Table 6, the theoretical color change range of our prepared sensitive membrane is 2.8~9.0. Actually, when the sensitive membrane was dipped into solutions with 2.5 < pH < 3.0 and 9.0 < pH < 11.0, the absorbance curves also changed, indicating that it can also work with pH values varying from 2.5 to 11.0, which may be due to the interaction of the four indicators.

In a neutral solution, the working indicators are mainly chlorophenol red and cresol red. However, the p*K*_a_ values of the two indicators are on the boundary of the best detecting range; thus, the color change of the indicators is not obvious, indicating that the sensor works worse when the solution is near to neutral, accounting for the results obtained in Figure 7 and Figure 10.

## 4. Conclusions

Making use of the sol–gel technique, an optical pH sensor was prepared, which was made of an organic carrier with four indicators (congo red, bromophenol blue, cresol red, and chlorophenol red) cross-linked by tetraethyl orthosilicate (TEOS) and cellulose acetate. The actual detection range of the sensor is between 2.5 and 11.0 with an accuracy of 0.2 pH. The optimal amounts of ethyl orthosilicate, absolute ethanol, deionized water, and hydrochloric acid in the glue precursor of the sensor-sensitive membrane were explored. The orthogonal test was performed to optimize the dosages of cellulose acetate, DMF, indicator, hydrochloric acid, and precursor glue. The performance parameters of linearity, measurement accuracy, repeatability, stability, and response time of the prepared pH sensors were tested.

The performance upon verifying the results after optimization of the preparation parameters shows that our optical pH sensor has high linearity in the pH buffer series of pH 2.5–11.0 with an accuracy of measurement up to 0.2 pH, good stability and repeatability, as well as a shorter response time compared with the traditional glass electrode. The predicted model based on full spectral data contained more useful information; thus, it has better accuracy than the single-wavelength model.

The optical pH sensor proposed in this paper has a wide working range, and it can probably withstand high temperatures and high pressure, compared with electric chemical electrodes. Therefore, it has an important application value in the fields of clinical medicine detection, biopharmaceutical analysis, water quality detection, etc.

## Figures and Tables

**Table 1:** The $L_{16}(4^{5})$ factor level of the pH-sensitive membrane composition ratio.

	Factors	A	B	C	D	E
Levels		Cellulose Acetate (g)	Mixed Indicator (g)	DMF (mL)	Hydrochloric Acid (µL)	Precursor (mL)
1		0.10	4	15	40	7
2		0.15	6	20	45	7.5
3		0.20	8	25	50	8
4		0.25	10	30	55	8.5

**Table 2:** Orthogonal test results of pH-sensitive membrane composition ratio.

Test Number	A	B	C	D	E	R
1	1	1	1	1	1	2.36
2	1	2	2	2	2	2.23
3	1	3	3	3	3	2.11
4	1	4	4	4	4	1.65
5	2	1	2	3	4	1.92
6	2	2	1	4	3	1.42
7	2	3	4	1	2	1.05
8	2	4	3	2	1	1.19
9	3	1	3	4	2	1.21
10	3	2	4	3	1	0.58
11	3	3	1	2	4	1.88
12	3	4	2	1	3	1.51
13	4	1	4	2	3	0.15
14	4	2	3	1	4	0.24
15	4	3	2	4	1	1.49
16	4	4	1	3	2	1.37
$k_{1}$	2.0875	1.41	1.7575	1.29	1.405	
$k_{2}$	1.395	1.1175	1.7875	1.3625	1.465	
$k_{3}$	1.295	1.6325	1.1875	1.495	1.2975	
$k_{4}$	0.8125	1.43	0.5875	1.4425	1.4225	

R = |Slope of the fitting line × 100%|.

**Table 3:** Power response of optimized pH sensor to pH standard buffer.

P_1_ (μW)	P_2_ (μW)	P_3_ (μW)	P (μW)	pH_buffer_	pH_t^heoretical^_	|offset|
8.21	8.21	8.20	8.206667	4.00	3.92	0.08
8.13	8.15	8.14	8.14	6.86	6.62	0.24
8.07	8.08	8.07	8.073333	9.18	9.31	0.13

**Table 4:** Standard deviations and relative standard deviations of measurements of different pH buffers.

pH Buffer	σ	$C_{V}$
4.0	0.00305505	0.37322%
6.0	0.002516611	0.309065%
8.0	0.007767453	0.959103%
10.0	0.003	0.372439%

**Table 5:** Average absolute error of pH spectral model and fit line.

Method	pH = 4.0	pH = 6.86	pH = 9.18
Power–pH fitting line	0.083	0.261	0.148
Absorbance–pH model	0.052	0.225	0.098

**Table 6:** Acid dissociation constant (p*K*_a_) of the four indicators.

Indicator	Congo Red	Bromophenol Blue	Cresol Red	Chlorophenol Red
p*K*_a_	4.1	3.85	8.0	6.0
